# Filter Pruning via Measuring Feature Map Information

**DOI:** 10.3390/s21196601

**Published:** 2021-10-02

**Authors:** Linsong Shao, Haorui Zuo, Jianlin Zhang, Zhiyong Xu, Jinzhen Yao, Zhixing Wang, Hong Li

**Affiliations:** 1 Key Laboratory of Optical Engineering, Chinese Academy of Sciences, Chengdu 610200, China; shaolinsong19@mails.ucas.ac.cn; 2 Institute of Optics and Electronics, Chinese Academy of Sciences, Chengdu 610200, China; jlin@ioe.ac.cn (J.Z.); xzy158@163.com (Z.X.); yaojinzhen19@mails.ucas.ac.cn (J.Y.); E190068@e.ntu.edu.sg (Z.W.); lihong19@mails.ucas.ac.cn (H.L.); 3 University of Chinese Academy of Sciences, Beijing 100049, China

**Keywords:** model compression, filter pruning, information entropy, normalization

## Abstract

Neural network pruning, an important method to reduce the computational complexity of deep models, can be well applied to devices with limited resources. However, most current methods focus on some kind of information about the filter itself to prune the network, rarely exploring the relationship between the feature maps and the filters. In this paper, two novel pruning methods are proposed. First, a new pruning method is proposed, which reflects the importance of filters by exploring the information in the feature maps. Based on the premise that the more information there is, more important the feature map is, the information entropy of feature maps is used to measure information, which is used to evaluate the importance of each filter in the current layer. Further, normalization is used to realize cross layer comparison. As a result, based on the method mentioned above, the network structure is efficiently pruned while its performance is well reserved. Second, we proposed a parallel pruning method using the combination of our pruning method above and slimming pruning method which has better results in terms of computational cost. Our methods perform better in terms of accuracy, parameters, and FLOPs compared to most advanced methods. On ImageNet, it is achieved 72.02% top1 accuracy for ResNet50 with merely 11.41M parameters and 1.12B FLOPs.For DenseNet40, it is obtained 94.04% accuracy with only 0.38M parameters and 110.72M FLOPs on CIFAR10, and our parallel pruning method makes the parameters and FLOPs are just 0.37M and 100.12M, respectively, with little loss of accuracy.

## 1. Introduction

With the development of deep neural networks in recent years, great success has been achieved in computer vision applications [1,2,3,4]. However, their apparent effectiveness is based on increasing storage, memory footprint, computational resources, and energy consumption, making most advanced Convolutional Neural Networks (CNNs) impossible to be deployed on edge devices such as cell phones and light devices. Although there are deep neural network acceleration frameworks such as TensorRT, they cannot reduce the network model. Therefore, there is still an important demand to reduce the parameters and floating point operations (FLOPs) of CNNs while keeping the accuracy unchanged. Common techniques include quantization [5,6,7,8], knowledge distillation [9,10,11], and network pruning [12,13,14,15,16]. In earlier work, pruning approaches [17,18] mainly used unstructured methods to obtain filters for irregular sparsity. To facilitate the deployment of models on general-purpose hardware and/or the use of basic linear algebra subroutine (BLAS) libraries, recent works have focused more on structured pruning or filter pruning [19,20,21], which simultaneously pursues the reduction of model size and improvement of computational efficiency.

The existing pruning methods are usually classified into two categories based on their compact CNN learning process: (1) Pretraining-dependency pruning, which is based on pretrained filter weights (e.g., $\ell_{1}$-norm [22] and coreset [23]) or data-driven activation such as output sparsity [24], rank of feature map [14] and the effect on accuracy or loss [25,26] of the intrinsic criteria measured with the aim of preserving important filters. (2) Regularization-retraining pruning, which introduces sparsity constraints [27,28,29] and masking schemes [30] during the training process. Although this method is very simple and eliminates the dependence on the pre training model, it usually needs to train from scratch, so the computational cost is very high. In addition, due to the introduction of sparse constraints, it brings great difficulties to the universality and flexibility of training loss.

In this paper, two novel pruning methods are proposed. First, we pay attention to the information of output feature maps, and propose a novel pruning method: directly calculate the information entropy of feature maps, and the importance of obtaining the corresponding filters (the richer the information of the feature maps, the more important the corresponding filters), to reduce the redundancy of the network filters. Moreover, we normalize the importance of feature maps of various layers to the same scale to avoid layer-by-layer pruning ratios. Secondly, we propose a new parallel pruning method by combining two methods including our first pruning method based on the entropy of the feature maps and Network Slimming [12]. Although above two pruning strategies have different advantages and effects, our proposed second parallel pruning has better effect by combining their advantages to make the network more compact.

We then compare the proposed methods with other advanced criteria. Experiments demonstrate that our methods can compress various CNN models consisting of VGGNet [1], DenseNet [31] and ResNet [32] on different image classification datasets such as CIFAR10/100 [33] and ImageNet [34]. The effectiveness of our methods is verified on several benchmarks, and our methods have better performance in terms of accuracy, parameters and the computational cost compared to the existing methods [12,14,15,16,20,21,22,35,36,37,38,39,40,41].

In the following, we will first discuss the related work in Section 2. Then, we elaborate our two pruning method in Section 3. In Section 4, the experimental results are provided and analyzed. Lastly, we conclude this paper in Section 5.

## 2. Related Work

### 2.1. Weight Pruning

Weight pruning removes individual neurons in the filter or connections between fully connected layers. Cun et al. [17] proposed the OBD (optical brain damage) algorithm which used loss to find the second order derivatives of the parameters to resolve the importance of parameters. Based on this, without limiting the diagonal assumption of the OBD algorithm, Hassibi [42] proposed the OBS (optical brain surgeon) algorithm, which recomputed other weights to compensate for the activation values in addition to setting the less important weights to 0, resulting in better compression effect. Similar to the OBS algorithm, Srinivas and Babu [43] proposed to remove the dense connections in the fully connected layer without relying on the training data, which greatly reduces the computational complexity. Recently, Dong et al. [44] proposed a layer-by-layer OBS algorithm, where each layer was independently pruned in terms of the second-order derivatives of the corresponding parameters of the layer-by-layer loss function, and after pruning, it was lightly retrained to recover the performance. In [45], 2-D DCT transformation is applied to sparsify the coefficients for spatial redundancy removal. Group sparsity-based regularization of network parameters [46] is leveraged to penalize unimportant parameters. Han et al. [18] introduced an iterative weight pruning method by fine-tuning with a strong $\ell_{2}$ regularization and discarding the small weights with values below a threshold. In [47], pruning and splicing are proposed to solve the problem that important filters may be removed in the pruning process, leading to a decrease in accuracy. Lin et al. [48] proposed a dynamic assignment of sparse patterns and the inclusion of feedback signals to reactivate the early pruned weights. However, weight pruning leads to irregular sparsity that requires special hardware/software, and this sparsity is difficult to support practical speedups on general-purpose devices [49].

### 2.2. Filter Pruning

In contrast, generic hardware and software can support filter pruning well, as it removes the entire filter without changing the original convolutional structure. For this purpose, Li et al. [22] used the importance of the filter based on the L1/L2 paradigm. Hu et al. [24] believed that channels with more sparse outputs are redundant and thus removed the corresponding filters and used the Average Percentage of Zeros (APoZ) as a metric based on the percentage of zeros in the activation layer. Luo and Wu [13] used the result of GAP of output feature map to obtain information entropy and remove redundant filters. Molchanov et al. [25] adopted Taylor expansion to approximate the influence to the loss function induced by removing each filter. Similarly, Yu et al. [38] optimized the reconstruction error of the final output response and propagates an “importance score” for each channel. He et al. [50] presented a LASSO-based filter selection strategy to identify representative filters and a least square reconstruction error to reconstruct the outputs. Luo et al. [41] established filter pruning as an optimization problem, and removed less important filters based on the statistics of the next layer. There was also a combination of various regularizers to make the weights of the network sparse. Lin et al. [36] used dynamic-coded filter fusion (DCFF) is introduced to train compact CNNs. Wen et al. [51] used Group Lasso for structured sparse. Huang and Wang [35] performed structured pruning by introducing learnable masks and using APG algorithm to sparse the masks. In [12], the scaling factor in the batch normalization(BN) layer is considered to be a filter selection indicator to decide whether a filter is important. However, the influence of shifting parameters in the BN layer is totally ignored [20]. Inspired by this, Kang and Han [52] considered both the channel scaling and shifting parameters for pruning. Lin et al. [14] observed the invariance of feature map rank and removed filers with low-rank feature maps. Yan et al. [15] combined $\ell_{1}$ Norm, number of parameters and computational effort as pruning criteria.

Please note that [13,14] investigated feature maps for network pruning. However, our guidelines for feature map evaluation are fundamentally different from [13,14]. First of all, Luo and Wu [13] performed global average pooling of feature maps followed by importance measures, and Lin et al. [14] used the rank of feature maps to determine importance, whereas we directly study the feature information contained in feature maps. Then, the methods used by Luo and Wu [13] loosened some information from the feature map because of global average pooling, which makes the importance measure of the filter inaccurate, while we study the complete feature map, which is richer in information obtained, and its importance measure of the filter is more accurate. At last, Lin et al. [14] required manually setting different pruning rates for each layer when pruning, while we only need to set a global pruning rate to achieve a good pruning effect.

## 3. The Proposed Method

**Figure 1:**
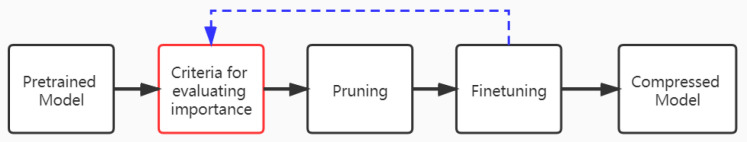
A typical pipeline of pruning.

**Figure 2:**
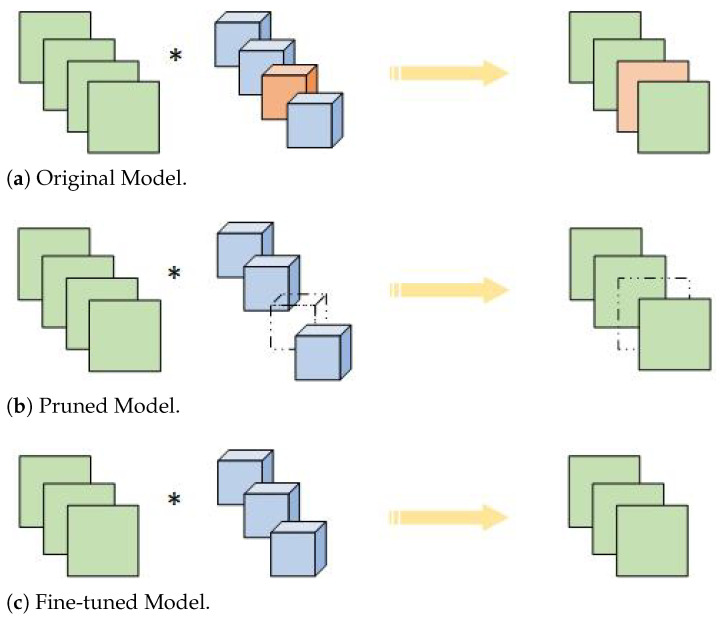
Illustration of Pruning Method. (1) The feature maps after the filters are focused on and its feature information is measured. (2) Feature information is positively correlated with the overall performance of its impact, and feature maps (dotted boxes) that contain less feature information are selected. (3) The associated filters (dashed cube) are discarded. (4) Finally, the pruning model is obtained.

The typical pipeline of a conventional pruning algorithm is shown in Figure 1, and has three steps: (1) the importance of the filter was calculated according to the evaluation criteria; (2) the importance values are sorted, and the model pruning ratio and the least important value determined under the condition of obtaining the ratio are specified; (3) the pruned model uses the original data for finetuning. Figure 2 illustrates the overall framework of our proposed feature map information pruning method. For the specific layer we want to prune, we first focus on its output feature map. If there is less feature map information, we have enough confidence that the corresponding filter is not so important, which could be pruned. In this paper, a new method based on information entropy is proposed to evaluate the feature map with less information. As shown in Figure 2, these feature maps with less information and the corresponding filters are highlighted in a dotted box.

### 3.1. Notations

Assume a pretrained CNN model has L layers, and $C^{l}$ (l∈[1,2,…,L]) is the *l*-th convolution layer. The shape of filters in $C^{l}$ is $W_{l}\in R^{N_{l}\times M_{l}\times K_{l}\times K_{l}}$, the number of input channels is $M_{l}$ in the $C^{l}$ layer. $N_{l}$ is both the number of output channels and the number of filters in the current convolution layer. $K_{l}\times K_{l}$ is the *l*-th convolutional kernel size. $X^{l}$ denotes the input of *l*-th layer and its shape is $I^{l}\times I^{l}\times M_{l}$ (the dimension of input feature maps of the *l*-th layer is $I^{l}\times I^{l}$). $Y^{l}$ denotes the output of *l*-th layer and its shape is $O^{l}\times O^{l}\times N_{l}$ (the dimension of output feature maps of the *l*-th layer is $O^{l}\times O^{l}$).

(1) $$Y_{k}^{l}=W_{lk}⊗X^{l},k=1,2,\ldots ,N_{l}$$

where $Y_{k}^{l}$ is the *k*-th channel of $Y^{l}$, ⊗ denotes the standard convolution operation, and $W_{lk}$ denotes the *k*-th filter of $W_{l}$.

The goal of filter pruning is to search a L-layers compact CNN model, where the filter shape of the *l*-th layer convolution $C^{l}$ is ${\tilde{W}}_{l}\in R^{{\tilde{N}}_{l}\times {\tilde{M}}_{l}\times K_{l}\times K_{l}}$ and ideally it should be satisfied that ${\tilde{N}}_{l}\leq N_{l}$. Then, the convolution in the *l*-th layer Equation (1) under the compact model framework can be reformulated as:

(2) $${\tilde{Y}}_{k}^{l}={\tilde{W}}_{lk}⊗{\tilde{X}}^{l},k=1,2,\ldots ,{\tilde{N}}_{l}$$

where ${\tilde{X}}^{l}$ and ${\tilde{Y}}^{l}$ denotes the input and output of *l*-th layer in the compact network, respectively.

### 3.2. Pruning Criteria

#### 3.2.1. Feature Maps Probability

To facilitate the calculation of the entropy value of output feature maps obtained by the filter, the feature maps should be processed, where it uses the softmax function. The feature maps probability obtained by convolution of the *l*-th layer use softmax function:

(3) $$s_{i}=\mathrm{softmax}\left(z_{i}\right)=\frac{e^{z_{i}}}{\sum_{j=1}^{J}e^{z_{j}}}$$

where $z_{i}$ represents the *i*-th pixel value of each feature map, and $s_{i}$ is the probability of the *i*-th position of the feature map. J denotes the total number of pixel values of the current feature map

When the information of feature map is richer, the pixel value at that location is different from the pixel value of the background. To highlight the obvious feature information, the improved softmax function is used as follows:

(4) $$\begin{array}{cc} s_{i} & =\frac{e^{v_{i}-v_{m}}}{\sum_{j=1}^{J}e^{v_{j}-v_{m}}}\\ v_{m} & =\max_{i}v_{i}\\ v_{i} & ={\left({z_{i}-\bar{z}}_{i}\right)}^{2}\\ \bar{z} & =\frac{1}{K}\sum_{i}z_{i} \end{array}$$

where *K* is the product of the dimension size of the feature map. Finally, the probability matrix S is obtained, where $s_{i}\in S$.

As can be seen from the content of Section 4.4, the improved softmax (I-Sofmax) has the effect of suppressing background information and highlighting local information compared to the conventional softmax (C-Softmax), which is beneficial to the entropy solution and makes the importance assessment criterion more accurate.

#### 3.2.2. Feature Maps Entropy

To calculate the entropy value corresponding to the filter, we first pass its feature map through the above improved softmax function Equation (4), and we obtain a probability matrix S. Finally, the entropy can be calculated as follows:

(5) $$E_{i}^{l}=-\sum_{k=1}^{K}s_{k}\log s_{k}$$

where $E_{i}^{l}$ denotes the entropy value of the *i*-th feature map in the *l*-th convolution layer. $s_{k}$ is the probability of the *k*-th position of S. K denotes the product of the dimension size of current feature map.

(6) $$\mathrm{Imp}\left(Y_{i}^{l}\right)=ES_{i}^{l}=\sum_{j=1}^{M}E_{ij}^{l}$$

where $\mathrm{Imp}\left(Y_{i}^{l}\right)$ and $ES_{i}^{l}$ are the importance evaluation score of $Y_{i}^{l}$ of the *l*-th layer. $ES_{i}^{l}$ is summed the entropy value obtained in each batch. $E_{ij}^{l}$ is the entropy value of the *j*-th batch and M denotes the batch size.

However, due to the different sizes of the feature map in different layer, the entropy value in different layers makes a big gap. To make all the layers in whole network comparable, max-min normalization is presented to quantify them in same scale. We normalize the importance distribution of each layer to align the correction distribution to [0,1], which can be formulated as:

(7) $$\begin{array}{cc} NE_{i}^{l} & =\frac{ES_{i}^{l}-ES_{a}^{l}}{ES_{b}^{l}-ES_{a}^{l}}\\ ES_{a}^{l} & =\min_{i}ES_{i}^{l}\\ ES_{b}^{l} & =\max_{i}ES_{i}^{l},a,b\in \left[1,N^{l}\right]\;\mathrm{and}\;a\neq b \end{array}$$

among the evaluation values of feature maps of $Y^{l}$, $ES_{a}^{l}$ is the smallest an $ES_{b}^{l}$ is the largest.

Based on the above description, We can define the final importance evaluation criteria of $Y_{i}^{l}$ from Equations (6) and (7) specifically as:

(8) $$\mathrm{Imp}\left(Y_{i}^{l}\right)=NE_{i}^{l}$$

### 3.3. Parallel Pruning Criteria

**Figure 3:**
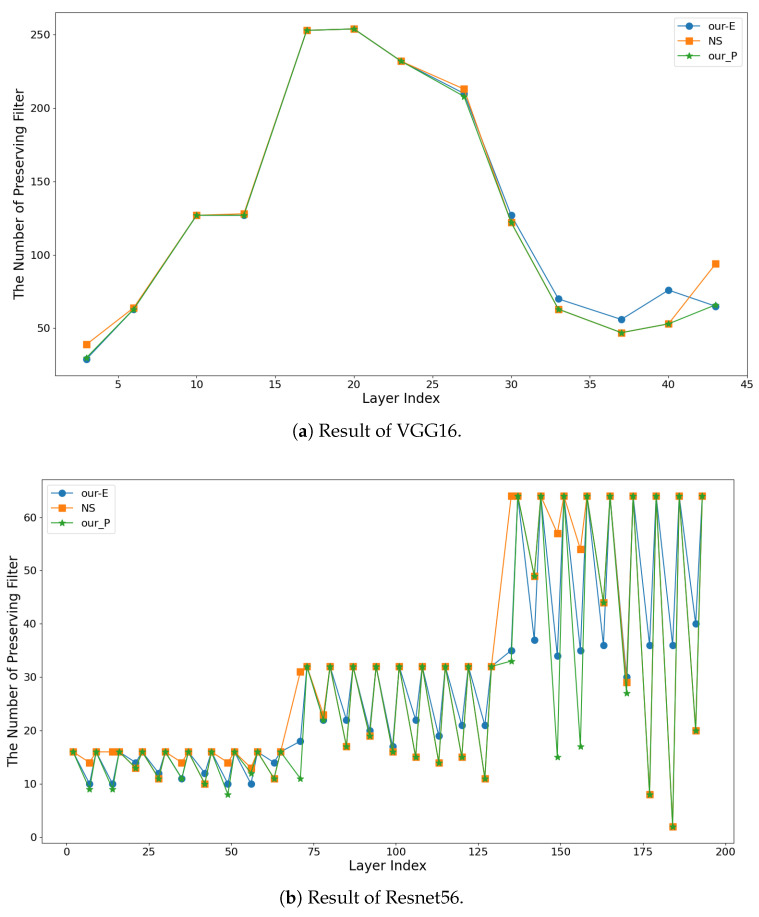
Results of VGG16 and Resnet56 on CIFAR10 using NS [12], Our-E (Algorithm 1) and Our-P (Algorithm 2) pruning methods at the same pruning rate.

We know that different metrics have different advantages and different pruning effects, resulting in different pruning rates for the same layer of the network. As can be seen in Figure 3, NS [12] pruning works well for pruning some layers of the network, while our pruning method based on the entropy of the feature maps (Algorithm 1) works well for other layers of the network. So we propose the parallel pruning method to apply both pruning algorithms to the same network value at the same time, and keep the best one of these two pruning results as the final result, as shown in our parallel pruning method (Algorithm 2) in Figure 3. This results in less parameters and FLOPs with little difference in accuracy.

To begin with, the network is trained to be sparse. It is trained to impose sparsity on the scale factor of the BN layer by L1 regularization, and then pruning is performed according to the size of the scale factor. Specifically, the optimization of the objective function is performed as follows.

(9) $$\hat{\theta }=\underset{\theta }{\arg\min}L\left(\theta \right)+\lambda \sum_{\gamma \in \Gamma }\left|\gamma \right|$$

where L is loss function and the latter term is a sparsity penalty term. γ is a scaling factor. A set of scaling factors in the neural network is Γ, while the degree of sparsity is controlled by λ.
**Algorithm 1** A Pruning Algorithm Based on Entropy of Feature Map1:**Input**: An L-layer Pre-trained CNN model with filter sets ${\left\{W_{l}\right\}}_{l=1}^{L}$, and the number of preserved filter in each layer ${\left\{{\tilde{N}}_{l}\right\}}_{l=1}^{L}$ and prune threhold δ.2:**Output**: The pruned network with filter sets ${\left\{{\tilde{W}}_{l}\right\}}_{l=1}^{L}$ and ${\tilde{W}}_{l}\in R^{{\tilde{N}}_{l}\times {\tilde{M}}_{l}\times K_{l}\times K_{l}}$.3:**while**l∈{1,2,…,L}**do**4:    Compute the feature maps Probability by Equation (4).5:    Obtain the importance scores for filters $W_{l}$ via Equation (7).6:    Get the preserved filer set ${\tilde{W}}_{l}$ by threhold δ and the importance scores.7:**end while**8:Get the pruned model without fine-tune.9:**while**t=1→T**do**10:    Fine-tune the pruned model.11:**end while**12:Return the pruned model with filter sets ${\left\{{\tilde{W}}_{l}\right\}}_{l=1}^{L}$.

**Algorithm 2** A Parallel Pruning Algorithm
**Require:** Training set $\left\{\left({\hat{x}}_{i},{\hat{y}}_{i}\right)\right\}$, and two threholds $\delta_{1}$ and $\delta_{2}$.
**Ensure:** The compat network.
1:
**while** t=1→T **do**
2:    The model is obtained by sparsity training through Equation (9)3:
**end while**
4:
**while** l∈{1,2,…,L} **do**
5:    mask1 = I ($\delta_{1}>NE^{l}$) by Algorithm 1;6:    mask2 = I ($\delta_{2}<\gamma$) and Equation (9);7:    **if** sum(mask1)<=sum(mask2) **then**8:        mask=mask1;9:    **else**10:        mask=mask2;11:    **end if**12:    Pruning the current layer ← mask;13:
**end while**
14:Obtain the compat model without fine-tuning.15:
**while** t=1→T **do**
16:    Fine-tune the compat model.17:
**end while**
18:Return the compat model


**Figure 4:**
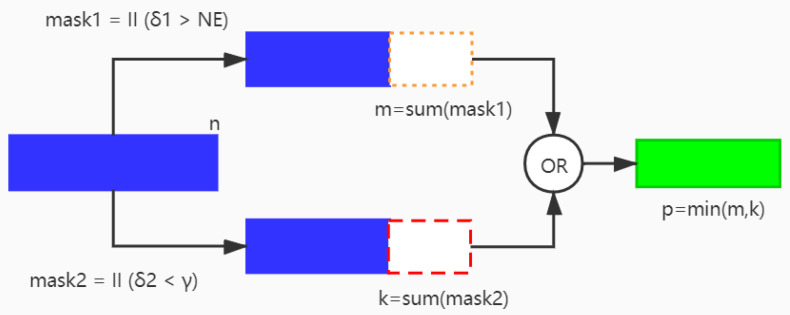
Schematic diagram of a parallel pruning algorithm. Prune a certain layer of the network, which contains n filters; m is the number of filters preserved by pruning according to the scaling factor; k the number of filters retained by our pruning method; p is the number of final filters reserved, which is the result of the minimum number of filters between m and k.

Then, the preserving filters are obtained by comparing the respective thresholds with the importance assessment values of filters. As shown in Figure 4, our proposed a parallel pruning method is used to prune one layer of the network, assuming that the layer contains n filters. The method mainly includes three steps: (1) According to our method based on the entropy of feature map (Algorithm 1), the importance values $NE^{l}$=($NE_{1}^{l}$,$NE_{2}^{l}$,…,$NE_{n}^{l}$) of the layer are obtained. Then, it is determined whether to retain the corresponding filter according to the threshold $\delta_{1}$. We use the indication function I ($\delta_{1}>NE^{l}$) to represent the determination process and obtain the result mask1. (2) At the meantime, according to NS method, we obtain the corresponding importance values γ=($\gamma_{1}$,$\gamma_{2}$,...,$\gamma_{n}$) of the layer, and the corresponding retention result mask2 are obtained according to the indicator function I ($\delta_{2}<\gamma$), where $\delta_{2}$ is a threshold and $\delta_{2}<\gamma_{k}\left(k\in \left[1,n\right]\right)$ means that the *k*-th filter of this layer is reserved. (3) The filter retention result mask of this layer is the smallest result in mask1 and mask2. As can be seen from Figure 4, m and k are the sizes of mask1 and mask2, respectively, and the final size p of mask is the smallest of m and k. The specific steps are described in Algorithm 2.

The experimental results of the parallel pruning method prove the effectiveness of the method, as described in Section 4.

### 3.4. Pruning Strategy

Traditional convolution structure and recent structural variants are two main architectures of the current network structure. The typical former is VGGNet [1], while the latter mainly contains several recent networks such as DenseNet [31] and ResNet [32].

**Figure 5:**
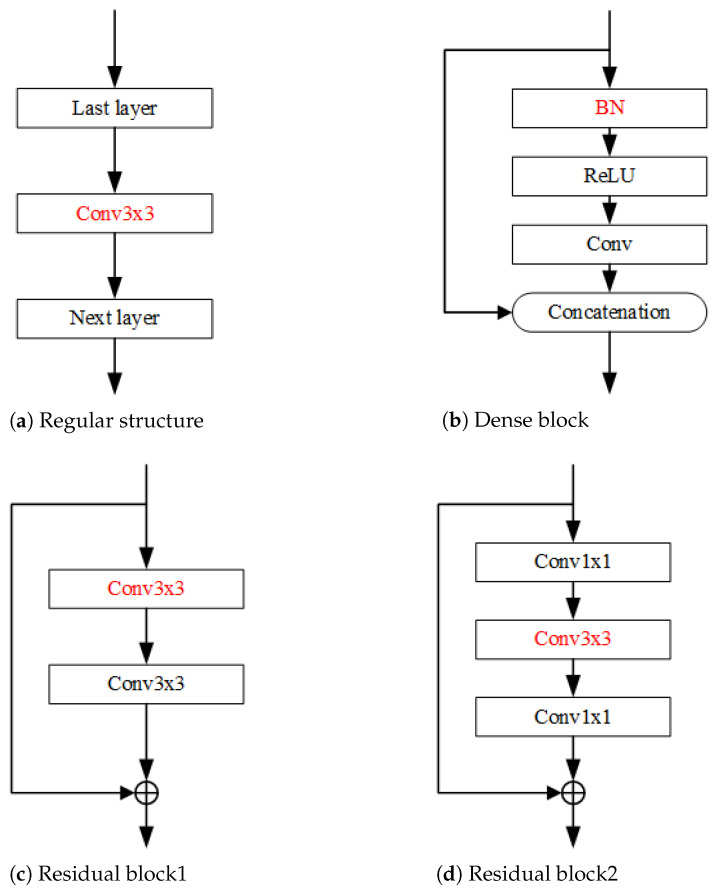
An illustration of mainstream network structure to be pruned, including Regular structure [1], Dense block [31] and Residual block [32].

These networks are pruned by different strategies. For VGGNet, they are all conventional convolutional layers with direct conventional pruning, where the importance of the feature maps obtained from each convolutional layer are evaluated and then pruned according to the importance threshold, as shown in Figure 5a.

For ResNet, there are some limitations owing to its special structure. For example, in order to complete the summation operation, the channel numbers of each block of the same group need to be consistent. Therefore, it is difficult to directly prune the last convolutional layer of each residual block. Like ResNet164 with three convolution layers per block, most parameters are located in the first two layers. Similarly, each block of ResNet56 has two layers, and most parameters are located in the first layer. Therefore, for each block of ResNet, it is a good choice to keep only the last layer and prune other layers, as shown in Figure 5c,d.

DenseNet also has a special structure with certain limitations. Due to the growth rate setting, each dense block generates the same number of feature maps, which are then fused with the previous feature maps. Therefore, it is difficult to directly evaluate the importance of the feature maps generated by the convolutional layers of each dense block. Since each dense block has a BN layer which is not affected by the growth rate, it is a good choice to evaluate the feature maps generated by the BN layer, which is illustrated in Figure 5b.

## 4. Experimental Results

To demonstrate the effectiveness and efficiency of our two proposed pruning methods (Algorithms 1 and 2), we conducted extensive experiments on image classification. Representative compact design networks, including VGG16/19 [1], Densenet40 [31], and ResNet50/56/164 [32], were chosen for compression and pruning. We report the performance of our two pruning methods on CIFAR10/100 [33] and ImageNet [34], and compare with the state-of-the-art (SOTA), our methods have great advantages. Please note that our method is different from the similar methods of Luo and Wu [13] and hrank [14], because we directly obtain the information contained in the feature maps through entropy, and we can set a global pruning rate to obtain a compact network.

### 4.1. Implementation Details

We carry out CIFAR experiments on NVIDIA RTX 2060 SUPER GPU and ImageNet experiments on NVIDIA RTX 3090 GPU. All models are implemented and trained using the deep learning framework Pytoch [53]. The effectiveness is validated on three datasets: CIFAR10, CIFAR100, and ImageNet. CIFAR10 includes images of 32 × 32 size from 10 classes. The training set includes 5000 images and the test set contains 10 k images. CIFAR100 includes images from 100 classes. Each class includes 600 pictures, divided into 500 training pictures and 100 test pictures. The ImageNet dataset composes of 1.28M training images and 50 k validation images, which are collected from 1 k categories.

All networks are trained using stochastic gradient descent (SGD), and we set weight decay and momentum to be $10^{-4}$ and 0.9, respectively. On CIFAR10 and CIFAR100, we train the networks for 160 epochs and set the batch size to 128. The initial learning rate is 0.1 and is multiplied by 0.1 at 50% and 75% of the total number of epochs. On ImageNet dataset, we used batch size of 256 to train the network for 160 epochs. The initial learning rate was 0.1, and then multiplied by 0.1 every 30 epochs.

### 4.2. Comparison on CIFAR10/100

As shown in Table 1 and Table 2, we analyzed on cifar10/100 through several popular networks, including VGG16/19, ResNet56/164, and DenseNet40. The classification accuracies of compressed models trained with our algorithm and the baseline method were compared. With similar accuracy, the method can effectively reduce parameters and FLOPs. This illustrates that our methods outperform existing pruning methods in reducing parameters.

**VGG16/19**. According to the results of VGG, it can be seen that our algorithm based on the entropy of the feature maps (Algorithm 1) has good results, for example, the compressed VGG16 achieves 93.53% accuracy with only 0.99M parameters and 83.96M FLOPs. using our parallel pruning method (Algorithm 2), the pruned VGG16 has less parameters and less FLOPs with little loss in accuracy. Therefore, our algorithm has the ability to compress the network to a more compact structure.

**ResNet56/164**. On CIFAR10, with similar parameters and FLOPs, our algorithm based on the entropy of the feature maps enables ResNet56 to obtain an accuracy of 93.56% with 0.39M parameters and 69.52M FLOPs, respectively. ResNet164 achieves an accuracy of 94.66% with 0.67M parameters and 111.33M FLOPs, respectively. In addition, using our parallel pruning algorithm is effective in reducing the computation with a slight loss of accuracy. Similarly, we can obtain the same results on CIFAR100. This shows that our algorithm is particularly suitable for pruning residual blocks.

**DenseNet40**. Our algorithm based on the entropy of the feature maps demonstrates that DenseNet40 can obtain 94.04% accuracy on CIFAR10 with only 0.38M parameters and 110.72M FLOPs. Meanwhile, it obtains 74.50% accuracy on CIFAR100 with only 0.40M parameters and 109.55M FLOPs. In addition, our parallel pruning method is able to obtain fewer parameters and computation with little difference in accuracy on both datasets. Overall, our algorithm has better results relative to existing algorithms, so it can work on networks with dense blocks, too.

**Figure 6:**
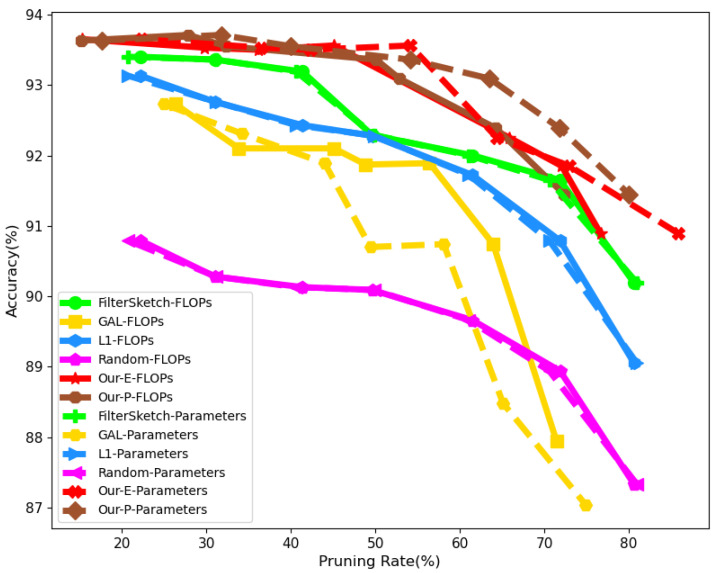
FLOPs and parameter comparison among GAL [21], L1 [22], Random, FilterSketch [16], and our two pruning methods under different compression rates, where Our-E and Our-P are Algorithms 1 and 2, respectively. ResNet56 is compressed and accuracy is reported.

In Figure 6, we further compare the accuracy of compressed models at different compression rates using ResNet-56 for GAL [21], L1 [22], Random, FilterSketch [16], and our two pruning methods (Algorithms 1 and 2). As shown in the figure, our two pruning methods easily outperform the compared methods. In particular, for larger pruning rates (>60%), the accuracy of L1, GAL and FilterSketch all show a great degradation, while our two algorithms maintain relatively stable performance, which emphasizes the importance of information preserving in network pruning again.

### 4.3. Comparison on ImageNet

The results of the comparative experiments on ResNet50 on ImageNet dataset are illustrated in Table 3. Overall, compared to existing methods, our method based on the entropy of the feature maps is superior to the most advanced method in every aspect, including top1 and top5 accuracy along with FLOPs and parameters reduction. To be more precise, ResNet50 achieves 72.02% accuracy with 11.41M parameters and 1.84B FLOPs, which is significantly better than HRank with 13.77M parameters and 1.55B FLOPs. In addition, our method based on the entropy of the feature maps goes further to obtain 70.41 top1 accuracy and 89.91 top5 accuracy with 8.51M parameters and 1.41B FLOPs. However, our parallel pruning method has a slight loss in accuracy compared to other methods. There is a slight shortfall in computational cost. In terms of the above results, Our approach has some processing power in complex datasets.

### 4.4. Ablation Study

**Figure 7:**
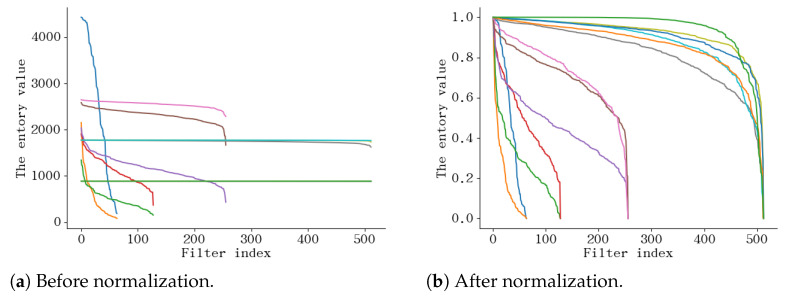
The entropy distribution of different layers calculated by VGG16 on CIFAR10. Different colors indicate different layers.

**Normalization**. From Figure 7a, we can see that the difference in entropy required by various layers is huge, so, to realize cross layer comparison, we normalize the values, as shown in Figure 7b. We tried and compared z-score normalization and max-min normalization with other settings held constant. In the final entropy evaluation, we decided to use max-min normalization. Table 4 illustrates the results on CIFAR10. We can see that reducing FLOPs and pruning more parameters with higher accuracy can be done using max-min normalization, which is the best choice.

**Figure 8:**
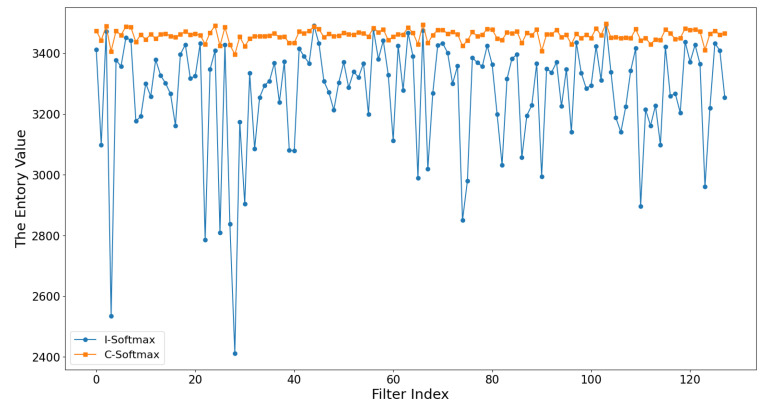
Entropy results of the feature map after the fourth layer of convolution of VGG16. The conventional softmax (C-Softmax) and the improved softmax (I-Softmax) produce different entropy distributions.

**Figure 9:**
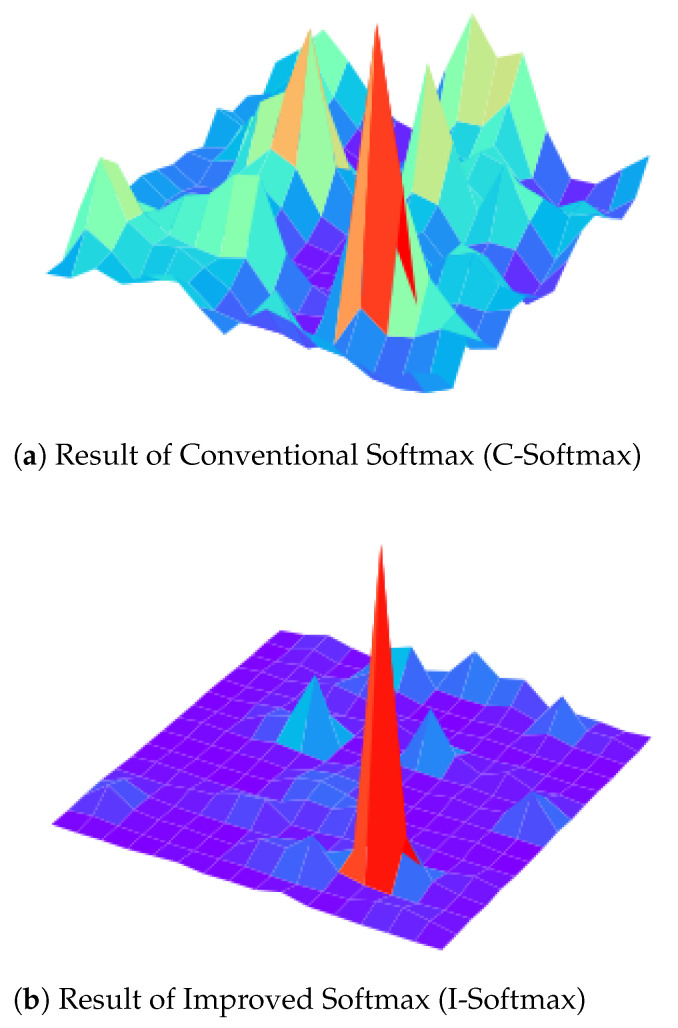
The visualization results of two softmax methods for feature maps. Compared to Conventional softmax Equation (3), Improved softmax Equation (4) has the effect of highlighting feature information and suppressing information such as background.

**Softmax**. To analyze the conventional softmax and the improved softmax, we conducted an analytical comparison. From Figure 8 we can see that the improved softmax facilitates a more decentralized distribution of entropy values compared to the conventional softmax, which is easier to achieve when evaluating the filter importance. In Figure 9, we visualize the feature maps after the two softmax methods, and we can see that Figure 9b can suppress the background and highlight the role of feature information than Figure 9a, which is beneficial to the subsequent entropy calculation. In Table 5, the implementation analysis on multiple networks further confirms that the improved softmax is more conducive to the pruning effect. For example, VGG16 achieves an accuracy of 93.53% using the improved softmax, while the conventional softmax causes a serious decrease in model accuracy. The analysis found that the conventional pruning would prune the deep convolution of the network more strongly, making the FLOPs much less, but the accuracy would be seriously affected. So we decided to use the improved softmax as the final choice.

## 5. Conclusions

In this paper, we proposed two novel pruning methods to train compact CNNs. First, our proposed pruning method based on the feature map information entropy acts directly on the feature map, where the accuracy can be well maintained by this information entropy as the filter importance evaluation criterion. Secondly, we further propose a parallel pruning method, which can eliminate the limitations of a single pruning method and significantly reduce the complexity of model. Finally, Our parallel pruning method can be extended by integrating more pruning methods to achieve parallelization and obtain a more compact network model. Numerous experiments have proved the superiority of our filter pruning method over the latest methods.

## Figures and Tables

**Table 1:** Pruning reasult on CIFAR10, where Our-E is our proposed pruning method based on the entropy of the feature map, and Our-P is our proposed parallel pruning method.

Model	Alg	Acc(%)	Param	FLOPs
VGG16	Baseline	93.90	14.72M	313.75M
	NS [12]	93.69	3.45M	199.66M
	L1 [22]	93.40	5.40M	206.00M
	SSS [35]	93.02	3.93M	183.13M
	GAL-0.05 [21]	92.03	3.36M	189.49M
	VCNNP [20]	93.18	3.92M	190.01M
	HRank [14]	92.34	2.64M	108.61M
	DCFF [36]	93.47	1.06M	72.77M
	CP MC [15]	93.40	1.04M	106.68M
	SWP [37]	92.85	1.08M	90.60M
	Our−E	93.53	0.99M	83.96M
	Our−P	93.47	0.93M	89.02M
	Our−P	93.16	0.90M	79.85M
VGG19	Baseline	93.68	20.04M	398.74M
	NS [12]	93.66	2.43M	208.54M
	Our−E	93.63	1.55M	129.21M
	Our−P	93.58	1.45M	127.44M
ResNet56	Baseline	93.22	0.85M	126.55M
	NS [12]	92.94	0.41M	64.94M
	L1 [22]	93.06	0.73M	90.90M
	NISP [38]	93.01	0.49M	81.00M
	GAL-0.6 [21]	92.98	0.75M	78.30M
	HRank [14]	93.17	0.49M	62.72M
	KSE (G = 4) [39]	93.23	0.43M	60M
	DCFF [36]	93.26	0.38M	55.84M
	KSE (G = 5) [39]	92.88	0.36M	50M
	FilterSketch [16]	93.19	0.50M	73.36M
	Our−E	93.56	0.39M	69.52M
	Our−P	93.36	0.39M	63.15M
	Our−P	93.09	0.31M	59.66M
ResNet164	Baseline	95.04	1.71M	254.50M
	NS [12]	94.73	1.10M	137.50M
	CP MC [15]	94.76	0.75M	144.02M
	Our−E	94.66	0.67M	111.33M
	Our−P	93.65	0.73M	105.86M
DenseNet40	Baseline	94.26	1.06M	290.13M
	GAL-0.01 [21]	94.9	0.67M	182.92M
	HRank [14]	94.24	0.66M	167.41M
	VCNNP [20]	93.16	0.42M	156.00M
	CP MC [15]	93.74	0.42M	121.73M
	KSE (G = 6) [39]	94.70	0.39M	115M
	NS [12]	94.09	0.40M	132.16M
	Our−E	94.04	0.38M	110.72M
	Our−P	93.75	0.37M	100.12M

**Table 2:** Pruning result on CIFAR100, where Our-E is our proposed pruning method based on the entropy of the feature map, and Our-P is our proposed parallel pruning method.

Model	Alg	Acc(%)	Param	FLOPs
VGG16	Baseline	73.80	14.77M	313.8M
	VCNNP [20]	73.33	9.14M	256.00M
	NS [12]	73.72	8.83M	274.00M
	CPGMI [54]	73.53	4.99M	198.20M
	CPMC [15]	73.01	4.80M	162.00M
	Our−E	73.17	4.94M	150.70M
	Our−E	73.06	4.05M	129.52M
	Our−P	73.17	4.09M	147.99M
VGG19	Baseline	73.81	20.08M	398.79M
	NS [12]	73.00	5.84M	274.36M
	Our−E	73.29	4.21M	183.69M
	Our−P	73.15	4.17M	195.77M
	Our−P	73.01	3.94M	180.51M
ResNet56	Baseline	71.77	0.86M	71.77M
	NS [12]	70.51	0.60M	62,82M
	Our−E	71.28	0.50M	80.48M
	Our−P	70.67	0.41M	69.88M
ResNet164	Baseline	76.74	1.73M	253.97M
	NS [12]	76.18	1.21M	123.50M
	CPMC [15]	77.22	0.96M	151.92M
	Our−E	76.28	0.94M	150.57M
	Our−P	75.27	0.94M	123.09M
DenseNet40	Baseline	74.37	1.11M	287.75M
	VCNNP [20]	72.19	0.65M	218.00M
	CPGMI [54]	73.84	0.66M	198.50M
	CPMC [15]	73.93	0.58M	155.24M
	NS [12]	73.87	0.55M	164.36M
	Our−E	74.50	0.40M	109.55M
	Our−E	73.74	0.34M	95.79M
	Our−P	74.26	0.39M	108.81M
	Our−P	73.62	0.34M	94.84M

**Table 3:** Pruning results of ResNet50 on ImageNet, where Our-E is our proposed pruning method based on the entropy of the feature map, and Our-P is our proposed parallel pruning method.

Model	Top-1%	Top-5%	FLOPs	Parameters
ResNet50 [41]	76.15	92.87	4.09B	25.50M
SSS-32 [35]	74.18	91.91	2.82B	18.60M
[50]	72.30	90.80	2.73B	-
GAL-0.5 [21]	71.95	90.94	2.33B	21.20M
HRank [14]	74.98	92.33	2.30B	16.15M
GDP-0.6 [40]	71.19	90.71	1.88B	-
GDP-0.5 [40]	69.58	90.14	1.57B	-
SSS-26 [35]	71.82	90.79	2.33B	15.60M
GAL-1 [21]	69.88	89.75	1.58B	14.67M
GAL-0.5-joint [21]	71.80	90.82	1.84B	19.31M
HRank [14]	71.98	91.01	1.55B	13.77M
ThiNet-50 [41]	68.42	88.30	1.10B	8.66M
GAL-1-joint [21]	69.31	89.12	1.11B	10.21M
HRank [14]	69.10	89.58	0.98B	8.27M
NS [12]	70.43	89.93	2.54B	18.33M
Our−E	72.02	90.69	1.84B	11.41M
Our−E	70.41	89.91	1.41B	8.51M
Our−P	69.91	89.46	1.70B	11.06M
Our−P	68.62	88.62	1.34B	8.23M

**Table 4:** Pruning results of using different normalization.

Model	Algorithm	Acc(%)	Param	FLOPs
VGG16	$\;{\mathrm{Our}}_{z-score}$	93.04	0.97M	69.06M
	$\;{\mathrm{Our}}_{max-min}$	93.53	0.99M	83.96M
ResNet164	$\;{\mathrm{Our}}_{z-score}$	94.55	0.90M	133.06M
	$\;{\mathrm{Our}}_{max-min}$	94.66	0.67M	111.33M
DenseNet40	$\;{\mathrm{Our}}_{z-score}$	93.83	0.37M	149.75M
	$\;{\mathrm{Our}}_{max-min}$	94.04	0.38M	110.72M

**Table 5:** Pruning results of using two softmax methods. Where C-softmax is the conventional softmax, I-softmax is the imporved softmax.

Model	Algorithm	Acc(%)	Param	FLOPs
VGG16	C-softmax	92.97	0.98M	65.38M
	I-softmax	93.53	0.99M	83.96M
VGG19	C-softmax	91.85	1.72M	63.64M
	I-softmax	93.63	1.55M	129.21M
ResNet56	C-softmax	93.21	0.45M	63.11M
	I-softmax	93.56	0.39M	69.52M
ResNet164	C-softmax	93.91	0.67M	112.73M
	I-softmax	94.66	0.67M	111.33M
DenseNet40	C-softmax	94.17	0.39M	118.01M
	I-softmax	94.04	0.38M	110.72M

## Data Availability

Not applicable.

## Abbreviations

The following abbreviations are used in this manuscript:

CNN/CNNs	Convolutional Neural Networks
FLOPs	floating point operations
BN	Batch Normalization
GAP	global average pooling
DNS	Dynamic Network Surgery
SNIP	single-hot network pruning
APoZ	Average Percentage of Zeros
APG	Accelerated Proximal Gradient
OBD	Optical brain damage
OBS	Optical brain surgeon
KD	Knowledge Distillation
DML	Deep mutual learning
SGD	Stochastic gradient descent
SSS	Sparse Structure Selection
VCNNP	Variational convolutional neural network pruning
GAL	generative adversarial learning
GDP	Gobal and Dynamic pruning
DCFF	dynamic-coded filter fusion
NS	Network Slimming
